# Incidence and Factors Influencing Locomotive Syndrome in Cancer Patients Living in the Community

**DOI:** 10.7759/cureus.91856

**Published:** 2025-09-08

**Authors:** Yoshiteru Akezaki, Eiji Nakata, Masato Kikuuchi, Yoshimi Katayama, Shinsuke Sugihara

**Affiliations:** 1 Division of Physical Therapy, Kochi Professional University of Rehabilitation, Tosa, JPN; 2 Department of Orthopaedic Surgery, Okayama University Hospital, Okayama, JPN; 3 Department of Rehabilitation Medicine, National Hospital Organization Shikoku Cancer Center, Matsuyama, JPN; 4 Department of Rehabilitation Medicine, Okayama University Hospital, Okayama, JPN

**Keywords:** cancer, chemotherapy, factor, locomotive syndrome, rehabilitation

## Abstract

Background

Investigating locomotive syndrome (LS) of cancer survivors in the community will help clarify the importance of rehabilitation for cancer survivors in the community and provide a basis for exploring effective interventions. The primary purpose of this study was to conduct a comparison of LS, fatigue, psychological problems, and physical activity in cancer survivors and those without cancer in the community. The secondary purpose was to analyze factors influencing LS in cancer patients.

Methods

The study involved 59 cancer patients undergoing chemotherapy at home and 59 people without cancer. The cancer patients were those undergoing chemotherapy as outpatients and constituted the cancer group. The non-cancer people were living in the community and constituted the non-cancer group.

Cancer and non-cancer groups were surveyed and measured for LS, fatigue, psychological problems, and physical activity. The cancer group was also surveyed for the duration of chemotherapy treatment and the presence or absence of bone metastases.

Results

The cancer group was significantly more likely than the non-cancer group to have LS stage 2, to have fatigue, and to have psychological problems. Fatigue and psychological problems were significantly associated with LS stage 2.

Conclusions

Cancer patients in the community need to be assessed regularly by healthcare providers and interventions should be made according to their condition.

## Introduction

Cancer survivors at home have functional limitations, reduced activities of daily living (ADLs), and poor mental health [1-3]. It is recommended that chemotherapy not be used in patients with solid tumors who have an Eastern Cooperative Oncology Group (ECOG) performance status score of 3 or higher [4]. Administration of chemotherapy to patients with low performance status has been shown to result in lower response rates, higher incidence of toxic effects, and shorter survival. Patients with reduced ADL may have difficulty administering chemotherapy and may have shorter survival.

Locomotive syndrome (LS) is a term proposed by the Japanese Orthopaedic Association (JOA) to describe a condition in which a person's physical abilities (mobility) to stand, walk, run, climb stairs, and perform other activities of daily living are impaired due to fragile bones, joints, muscles, and other musculoskeletal organs [5,6]. Sarcopenia and frailty have complications of motor function, and LS assessing decline in mobility function represented by motor function may be a precursor to sarcopenia and frailty [7,8]. The progression of LS also increases the risk that the elderly and others will need long-term care services in the near future due to the functional deterioration in the locomotive organs [9]. Early detection of LS and subsequent countermeasures are expected to help improve quality of life, promote independence in ADLs, and reduce the need for nursing care [9].

A higher proportion of community-dwelling individuals without cancer also have LS as they age, but even those aged 20-64 years have locomotion [10]. Cancer survivors living in the community who are undergoing chemotherapy are expected to have further complications of LS due to the side effects of chemotherapy. Investigating LS of cancer survivors in the community will help clarify the importance of rehabilitation for cancer survivors in the community and provide a basis for exploring effective interventions.

The primary purpose of this study was to conduct a comparison of LS, fatigue, psychological problems, and physical activity in cancer survivors and those without cancer in the community. The secondary purpose was to analyze factors influencing LS in cancer patients.

## Materials and methods

Study design

This is a prospective study to compare cancer survivors and those without cancer in the community and to analyze factors affecting LS.

Patients

The study involved 59 cancer patients undergoing chemotherapy at home and 59 people without cancer in November 2020. The cancer patients were those undergoing chemotherapy as outpatients and constituted the cancer group. The non-cancer people were living in the community and constituted the non-cancer group. The non-cancer group was adjusted to match the cancer group for age and sex.

Exclusion criteria included those with clear impairments in ADLs, such as cerebral infarction, amputation, or spinal cord injury.

This study was approved by the Ethics Committee of Kochi Rehabilitation University (R1-14).

Methods

Cancer and non-cancer groups were surveyed and measured for LS, fatigue, psychological problems, and physical activity. The cancer group was also surveyed for the duration of chemotherapy treatment and the presence or absence of bone metastases. The cancer group was evaluated during the period of chemotherapy.

Locomotive syndrome

LS status was evaluated using the Locomo 25 score. The Locomo 25 includes four questions about pain, 16 questions about ADL, three questions about social function, and two questions about mental health status during the last month. The 25 items are scored on a 5-point scale from 0 (no impairment) to 4 (severe impairment), with higher total scores (ranging from 0 to 100) indicating more impaired locomotive function [11]. A Locomo-25 total score of 7 - 15 is classified as LS stage 1, a score of 16 - 23 as LS stage 2, and a score of ≥ 24 as LS stage 3. These stages represent stage 1 as the beginning of a decline in mobility, stage 2 as a progressive decline in mobility, and stage 3 as a significant decline that interferes with social participation [12]. 

In this study, the subjects were classified into “LS stage 0 and stage 1 group" and “LS stage 2 group”.

Fatigue

The Brief Fatigue Inventory was used for fatigue. The BFI has nine questions and is measured on a 0-10 numerical rating scale [13]. The first three questions assess current, usual, and worst levels of fatigue over the past 24 hours, with 0 being “no fatigue” and 10 being “fatigue as bad as you can imagine”. The interference items of six questions include general activity, mood, walking ability, normal work (includes both work outside the home and housework), relations with other people, and enjoyment of life. For interference items, 0 is “does not interfere” and 10 is “completely interferes”. The overall BFI score can be calculated by taking the average of the nine questions. Higher scores indicate higher levels of fatigue. The BFI classifies fatigue as no fatigue (0 points), mild fatigue (1 - 3 points), moderate fatigue (4 - 6 points), and severe fatigue (7 - 10 points) according to the total score. In this study, a score of 0 point was considered the no fatigue group, and a score of ≧1 was considered the fatigue group.

Psychological problems

The Distress and Impact Thermometer (DIT) is a self-administered questionnaire that screens for screening adjustment disorders and depression [14]. The DIT consists of two types of questions about the severity of the patient’s distress and impact. Each of the “distress” and “affect” questions is scored using an 11-point Likert scale, with scores ranging from 0 to 10, with higher scores indicating an unfavorable status.

The standard cut-off score for screening for adjustment disorder and major depression is a ‘distress’ score of four or above and ‘impact’ score of three or above. In this study, DIT was used to classify patients with adjustment disorders and depression into the psychological problem and non-psychological problem groups.

Physical activity

Physical activity was assessed using the International Physical Activity Questionnaire Short Form (IPAQ-SF). The IPAQ-SF has been used as an assessment of physical activity in several studies [15]. The IPAQ uses seven questions to assess the number of minutes spent in vigorous and moderate activity and walking during the last seven days and sitting on weekdays in the past seven days. In this study, an overall physical activity score (kcal) was calculated using a known formula for the IPAQ's sum of vigorous and moderate activity and walking.

Statistical analysis

Comparisons of gender, age, locomotor activity, fatigue, psychological problems, and physical activity between cancer and non-cancer groups were analyzed using Student's t-test, Fisher's exact test, and Mann-Whitney.

For the analysis of factors strongly influencing LS stage 2, univariate analysis was first carried out using Student’s t-test, Fisher's exact test, and the Mann-Whitney U test to extract the affective associated factors on LS stage 2. Next, for items with p <0.1 on univariate analysis, logistic regression analysis was used to identify the best independent predictor of LS stage 2. IBM SPSS Statistics for Windows, Version 22 (Released 2013; IBM Corp., Armonk, New York, United States). was used to analyze the data. The results were defined as being significant when the probability of error (p) was less than 5%.

Calculation of sample size was performed using G*Power (version 3.1.9.4), with alpha = 0.05 and power = 0.9. The number of patients was set at 59 per group, foreseeing a sample loss of around 10%.

## Results

Comparison between cancer and non-cancer groups

Characteristics of cancer groups are shown in Table 1.　

**Table 1:** Characteristics of the cancer group a) Mean ± standard deviation

Variable	
Cancer type (n)	
Breast cancer	21
Stomach cancer	9
Colorectal cancer	8
Lung cancer	8
Pancreatic cancer	4
Esophageal cancer	2
Prostate cancer	2
Multiple myeloma	2
Small intestine cancer	1
Ureteral cancer	1
Non-Hodgkin's lymphoma	1
Duration of chemotherapy treatment (day)^a)^	858.8±979.1
Bone metastases (n)	
Presence	21
Absence	38
Stage (n)	
Ⅰ	1
Ⅱ	2
Ⅲ	9
Ⅳ	47
Comorbidities (n)	
Hypertension	28
Diabetes	20
Osteoporosis	12
Heart disease	10
Dyslipidemia	5
Lung disease	4

Table 2 shows the results of univariate analysis for the cancer and noncancer groups. There were no significant differences in age and sex between the cancer and non-cancer groups. BMI was significantly lower in the cancer group than in the non-cancer group. The LS stage 2 group was significantly larger than the cancer group and the non-cancer group, 24 and 12, respectively (p<0.05). The fatigue group was significantly higher in the cancer group, with 34 and 18 patients in the cancer and non-cancer groups, respectively, (p<0.05). The psychological problem group was significantly higher in the cancer group, with 16 and six patients in the cancer and non-cancer groups, respectively, (p<0.05).

**Table 2:** Comparison of variables between the cancer and non-cancer groups LS: Locomotive syndrome a) Mean ± standard deviation

Variable	Cancer group (n=59）	Non-cancer group (n=59)	p-value
Age (y)^a)^	63.7±10.3	64.0±12.7	0.836
Sex (n)			0.573
Male	26	26	
Female	33	33	
Body mass index (kg/m^2)^	22.4±3.7	24.0±4.0	0.017
LS (n)			0.014
LS stage 2 group	24	12	
LS stage 0 and stage 1 group	35	47	
Fatigue (n)			0.003
Fatigue group	34	18	
No fatigue group	25	41	
Psychological conditions (n)			0.016
Psychological problem group	16	6	
Non-psychological problem group	43	53	
Physical activity^ a)^	362.8±316.2	274.2±421.3	0.199

Factors affecting LS stage 2

The results of the univariate analysis of the LS stage 2 and LS stage 0 and stage 1 groups are shown in Table 3. Fatigue, psychological problems, physical activity, duration of chemotherapy treatment, and the presence of bone metastases were different between the two groups (p<0.1). Logistic regression analysis showed that fatigue and psychological problems were significantly associated with LS stage 2 (p<0.05) (Table 4).

**Table 3:** Comparison of variables between the locomotive syndrome stage 2 and stage 0 and stage 1 groups a) Mean ± standard deviation LS: Locomotive syndrome

Variable	LS stage 2 group (n=24)	LS stage 0 and stage 1 group (n=35)	p-value
Age (y)^ a)^	65.4±9.0	62.6±11.1	0.303
Sex (n)			
Male	10	16	
Female	14	19	
Body mass index (kg/m^2^)^a)^	22.6±4.2	22.4±3.3	0.812
Fatigue(n)			P<0.0001
Fatigue group	21	13	
No fatigue group	3	22	
Psychological conditions (n)			P<0.0001
Psychological problem group	13	3	
Non-psychological problem group	11	32	
Physical activity^ a)^	448.8±314.8	303.8±307.8	0.042
Duration of chemotherapy treatment (days)^ a)^	1120.9±1007.6	679.2±931.0	0.038
Bone metastases (n)			0.026
Presence	13	8	
Absence	11	27	

**Table 4:** Factors predicting the locomotive syndrome: logistic regression analyses CI: Confidence interval.

Variable	Odds ratio (95% CI)	p-value
Fatigue	9.773(1.945-49.107)	0.006
Psychological conditions	7.565(1.389-41.191)	0.019
Physical activity	1.001 (0.998–1.003)	0.651
Duration of chemotherapy treatment	1.000(0.999-1.001)	0.855
Presence or absence of bone metastases	2.598(0.548-12.318)	0.229

## Discussion

The purpose of this study was to conduct a comparison of LS, fatigue, psychological problems, and physical activity in the cancer group and the non-cancer group in the community. The cancer group was significantly more likely than the non-cancer group to have LS stage 2, to have fatigue, and to have psychological problems.

The rate of LS stage 2 was about 20% for the non-cancer group, while the cancer group had about 40%, about twice the incidence. Studies of middle-aged and older adults aged 45 years and older have reported that cancer patients experience ADL limitation [1,2,16]. Few studies have examined the incidence of locomotion in cancer patients, so comparisons cannot be made. However, this study did find that the cancer group had more LS stage 2, or progressive loss of mobility, than the non-cancer group, and that more cancer patients at home were experiencing a decline in ADLs. A certain level of ADL is required to administer chemotherapy, and a decline in ADL may interrupt chemotherapy. Interruption of chemotherapy leads to decreased life expectancy. Therefore, cancer survivors in the community during chemotherapy need interventions to improve ADLs.

Cancer survivors are prone to fatigue and depression caused by the side effects of chemotherapy [17]. The study showed that significantly more cancer group had fatigue and psychological problems than non-cancer group. Cancer-related fatigue and depression were reported to occur in 60-100% [18-21] and 13.7% [22], respectively, of patients who were receiving chemotherapy. In the present study, cancer patients had fatigue in about 58% and mental problems in about 27%. Therefore, the results of the present study were similar to those of previous studies.

BMI was significantly lower in the cancer group compared to the non-cancer group. There was also no significant difference in the amount of physical activity between cancer patients and non-cancer patients. Since cancer patients may experience anorexia and weight loss due to chemotherapy, the decrease in BMI is thought to be an effect of chemotherapy rather than an increase in physical activity. A decrease in BMI has been reported to be associated with shorter overall survival [23,24]. In this study, the mean for the cancer group was 22 kg/m^2^, which was within the standard range. However, cancer patients may experience greater weight loss in the future, and nutritional interventions should be considered.

In a study of older cancer patients, patients with moderate to severe fatigue had a strong impact on IADL and ADL impairment [25]. This study found that fatigue and psychological problems were the strongest factors influencing LS stage 2 cancer patients during chemotherapy. It has been reported that fatigue and depression in cancer patients can be reduced by exercise [26,27]. Since this study is not an intervention study, the intervention effects on fatigue and mental problems cannot be determined. However, regular assessment of fatigue and mental problems and intervention including exercise for improvement may prevent or improve ADL decline.

Study limitations

First, physical function affects ADLs, but it is not clear to what extent physical function affects the degree of locomotion, since subjects are not assessed for physical function, such as muscle strength and balance function. Second, physical activity can be assessed with pedometers and other devices to evaluate physical activity in detail. In the present study, the amount of physical activity was evaluated by IPAQ, but it is also necessary to evaluate and analyze the amount of physical activity in more detail by using a pedometer in combination with the IPAQ. Third, bone metastasis was evaluated for the presence or absence of bone metastasis, but the degree of bone metastasis was not evaluated in detail. Fourth, differences based on cancer type or type of chemotherapy were not examined. Further study is needed in the future.

## Conclusions

The cancer group in the community who are treated with chemotherapy were significantly more likely to have LS stage 2, fatigue, psychological problems, and lower BMI than the non-cancer group. Cancer patients in the community need to be assessed regularly by health care providers and interventions should be made according to their condition. Fatigue and psychological problems also had a significant impact on LS stage 2. Therefore, to improve LS, interventions should focus not only on physical function but also on fatigue and psychological problems.
